# Supplementary material for: Digital Biohacking Approach to Dietary Interventions: A Comprehensive Strategy for Healthy and Sustainable Weight Loss
Source: Nutrients. 2024 Jun 26;16(13):2021. doi: 10.3390/nu16132021 (PMC11243021; doi:10.3390/nu16132021)
Supplement: Supplementary file 1 [file nutrients-16-02021-s001.zip › nutrients-2993725-supplementary.pdf]

nutrients

Supplementary Materials

Digital biohacking approach on dietary interventions: A Comprehensive Strategy for Healthy and Sustainable Weight Loss

Alessio Abeltino [1,2], Giada Bianchetti [1,2], Cassandra Serantoni [1,2], Alessia Riente [1,2], Marco De Spirito [1,2] and Giuseppe Maulucci [1,2,*]

[1] Dipartimento di Neuroscienze, Sezione di Biofisica, Università Cattolica del Sacro Cuore, Rome, Italy
[2] Fondazione Policlinico Universitario A. Gemelli IRCSS, Rome, Italy
* Corresponding author, mailing address, +39-06-3015-4265, and giuseppe.maulucci@unicatt.it

S1. Carbon footprint impact

The carbon footprint is a measure of the total greenhouse gas emissions, expressed in units of carbon dioxide equivalent (CO_2eq), that are directly or indirectly caused by an individual, organization, product, or activity [1]. When it comes to foods, it refers to the amount of greenhouse gas emissions associated with the production, processing, transportation, and disposal of food items throughout their entire life cycle, and it is estimated as a quarter of the total emission (Figure S2).

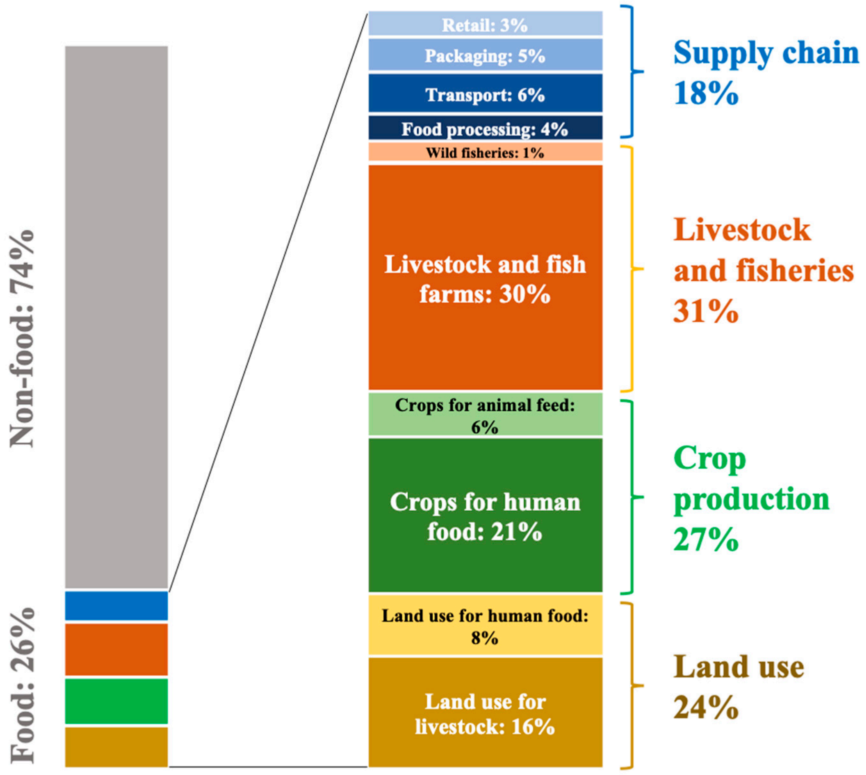

Figure S1. The figure shows the reported food production impact on the environment with the following stages: supply chain, livestock and fisheries, crop production, and land use. These data are retrieved from Our World in Data (https://ourworldindata.org/food-ghg-emissions, accessed on 25 January 2024).

This includes emissions from various stages such as agricultural practices, land use changes, energy consumption, transportation, packaging, and waste management.

Understanding the carbon footprint of different foods is essential for making informed choices that can help reduce the environmental impact of our diets. By opting for sustainably produced foods, reducing food waste, choosing plant-based options, supporting local and seasonal foods, and being mindful of energy use and transportation, individuals can make a positive impact in reducing the carbon footprint associated with their food consumption.

S2. Carbon footprint association

To associate a carbon footprint impact to all foods consumed by users, we used the My Emission free calculator as reference. We considered 431 generic foods divided into 17 macro categories listed in Table S1.

Table S1. Carbon footprint categorization[1].

Macro category	CO2 equivalent for 100 grams
Bakery foods	263 ± 223 gCO_2eq
Biscuits and confectionery	341 ± 182 gCO_2eq
Cupboard foods	155 ± 129 gCO_2eq
Dairy and alternatives	474 ± 275 gCO_2eq
Drinks	146 ± 73 gCO_2eq
Eggs	451 gCO_2eq
Fish and seafood	579 ± 471 gCO_2eq
Fruits	269 ± 167 gCO_2eq
Grains, pulses, and legumes	116 ± 68 gCO_2eq
Meat and alternatives	1025 ± 1240 gCO_2eq
Nuts and seeds	254 ± 122 gCO_2eq
Oils	290 ± 142 gCO_2eq
Potatoes	127 gCO_2eq
Processed foods	301 ± 118 gCO_2eq
Sauces and dressings	122 ± 137 gCO_2eq
Spreads, jams and honeys	187 ± 121 gCO_2eq
Vegetables	192 ± 125 gCO_2eq

[1] In the table, food macro-categories are listed with their own average carbon footprint impact. This data is retrieved from My Emission free calculator (https://myemissions.green/food-carbon-footprint-calculator/, accessed on 25 January 2024).

We have then made an association of this database with the diet lists of each user. Finally, we have calculated the relative carbon footprint for the quantitative reported on the list.

S3. Biohacking dictionaries

In this section, we present a comprehensive overview of the biohacking dictionaries created specifically for the four participants. The relevant information is presented in the following tables: Table S2, Table S3, Table S4, and Table S5.

Table S2. Biohacking dictionary for participant 0[1].

Food to change	Alternative	*Δ*intake	*Δ*carbon footprint	Meal	Quantity
Whole grain bread	Pancake	-15 kcal	-19.5 gCO_2eq	Breakfast	50 g
Rainbow trout	Codfish	-70.8 kcal	-121.2 gCO_2eq	Lunch	120 g
Sea bream	Codfish	-96.8 kcal	-126.5 gCO_2eq	Lunch	110 g
Wholemeal bread	Rye bread	-15.2 kcal	-11.2 gCO_2eq	Dinner	80 g
Low-alcohol bitters	Red wine	-123.2 kcal	-66.5 gCO_2eq	Dinner	70 g

[1] In this table, we present an overview of the biohacking dictionary for participant 0. The table provides information on variations in intake and carbon footprint, which are measured using the same quantity (indicated in the "Quantity" column) for the different types of foods.

Table S3. Biohacking dictionary for participant 1[1].

Food to change	Alternative	*Δ*intake	*Δ*carbon footprint	Meal	Quantity
Cherry croissant	Strudel	-75 kcal	-352 gCO_2eq	Breakfast	50 g
Chickpeas	Sweet corn	-408.8 kcal	-49 gCO_2eq	Lunch	140 g
Beefsteak	Chicken breast	-102.6 kcal	-6952 gCO_2eq	Lunch	180 g
Banana	Kiwi	-42 kcal	-102 gCO_2eq	Snack	150 g
Bresaola	Baked ham	-12.3 kcal	-3476 gCO_2eq	Dinner	95 g

[1] In this table, we present an overview of the biohacking dictionary for participant 1. The table provides information on variations in intake and carbon footprint, which are measured using the same quantity (indicated in the "Quantity" column) for the different types of foods.

Table S4. Biohacking dictionary for participant 2[1].

Food to change	Alternative	*Δ*intake	*Δ*carbon footprint	Meal	Quantity
Croissant	Lemon cake	-66.5 kcal	-176 gCO_2eq	Breakfast	50 g
Tuna in Olive Oil	Norwegian smoked salmon	-131 kcal	-301 gCO_2eq	Lunch	56 g
Eggplant	Mixed mushrooms	-196 kcal	-340 gCO_2eq	Lunch	200 g
Oro ciok	Pavesini	-27 kcal	-35 gCO_2eq	Snack	25 g
Seasoned Asiago DOP cheese	Sheep cheese	-5 kcal	-50 gCO_2eq	Dinner	30 g

[1] In this table, we present an overview of the biohacking dictionary for participant 2. The table provides information on variations in intake and carbon footprint, which are measured using the same quantity (indicated in the "Quantity" column) for the different types of foods.

Table S5. Biohacking dictionary for participant 3[1].

Food to change	Alternative	*Δ*intake	*Δ*carbon footprint	Meal	Quantity
Melba toasts	Biscuits	-51.1 kcal	-77.7 gCO_2eq	Breakfast	70 g

Turkey escalope	Chicken	-24 kcal	-174 gCO_2eq	Lunch	100 g
Fresh pasta	Rice	-34.5 kcal	-3 gCO_2eq	Lunch	150 g
Almonds	Pistachios	-2.34 kcal	-9.54 gCO_2eq	Snack	18 g
Hamburger	Chicken breast	-168 kcal	-5793 gCO_2eq	Dinner	150 g

[1] In this table, we present an overview of the biohacking dictionary for participant 3. The table provides information on variations in intake and carbon footprint, which are measured using the same quantity (indicated in the "Quantity" column) for the different types of foods.

References

1. Durojaye, O.; Laseinde, T.; Oluwafemi, I. A Descriptive Review of Carbon Footprint. In *Human Systems Engineering and Design II*; Springer: Berlin/Heidelberg, Germany, 2020; pp. 960–968. http://dx.doi.org/10.1007/978-3-030-27928-8_144.
